# Cardiac Oxidative Signaling and Physiological Hypertrophy in the Na/K-ATPase α1^s/s^α2^s/s^ Mouse Model of High Affinity for Cardiotonic Steroids

**DOI:** 10.3390/ijms22073462

**Published:** 2021-03-27

**Authors:** Pauline V. Marck, Marco T. Pessoa, Yunhui Xu, Laura C. Kutz, Dominic M. Collins, Yanling Yan, Cierra King, Xiaoliang Wang, Qiming Duan, Liquan Cai, Jeffrey X. Xie, Jerry B. Lingrel, Zijian Xie, Jiang Tian, Sandrine V. Pierre

**Affiliations:** 1 Marshall Institute for Interdisciplinary Research, Huntington, WV 25703, USA; pmarck14@gmail.com (P.V.M.); correapessoa@marshall.edu (M.T.P.); xuy@marshall.edu (Y.X.); kutz@live.marshall.edu (L.C.K.); collins375@live.marshall.edu (D.M.C.); king399@live.marshall.edu (C.K.); wangxi@marshall.edu (X.W.); cail@marshall.edu (L.C.); xiez@marshall.edu (Z.X.); tianj@marshall.edu (J.T.); 2 Department of Biomedical Sciences, Marshall University Joan C. Edwards School of Medicine, Huntington, WV 25755, USA; yan@marshall.edu; 3 Gladstone Institute of Cardiovascular Disease, San Francisco, CA 94158, USA; qiming.duan@rockets.utoledo.edu; 4 Department of Internal Medicine, University of Michigan, Ann Arbor, MI 48109, USA; xieje24@gmail.com; 5 Department of Molecular Genetics, Biochemistry and Microbiology, University of Cincinnati College of Medicine, Cincinnati, OH 45267, USA; lingrejb@ucmail.uc.edu

**Keywords:** Na/K-ATPase, cardiotonic steroids, isoform, reactive oxygen species, hypertrophy

## Abstract

The Na/K-ATPase is the specific receptor for cardiotonic steroids (CTS) such as ouabain and digoxin. At pharmacological concentrations used in the treatment of cardiac conditions, CTS inhibit the ion-pumping function of Na/K-ATPase. At much lower concentrations, in the range of those reported for endogenous CTS in the blood, they stimulate hypertrophic growth of cultured cardiac myocytes through initiation of a Na/K-ATPase-mediated and reactive oxygen species (ROS)-dependent signaling. To examine a possible effect of endogenous concentrations of CTS on cardiac structure and function in vivo, we compared mice expressing the naturally resistant Na/K-ATPase α1 and age-matched mice genetically engineered to express a mutated Na/K-ATPase α1 with high affinity for CTS. In this model, total cardiac Na/K-ATPase activity, α1, α2, and β1 protein content remained unchanged, and the cardiac Na/K-ATPase dose–response curve to ouabain shifted to the left as expected. In males aged 3–6 months, increased α1 sensitivity to CTS resulted in a significant increase in cardiac carbonylated protein content, suggesting that ROS production was elevated. A moderate but significant increase of about 15% of the heart-weight-to-tibia-length ratio accompanied by an increase in the myocyte cross-sectional area was detected. Echocardiographic analyses did not reveal any change in cardiac function, and there was no fibrosis or re-expression of the fetal gene program. RNA sequencing analysis indicated that pathways related to energy metabolism were upregulated, while those related to extracellular matrix organization were downregulated. Consistent with a functional role of the latter, an angiotensin-II challenge that triggered fibrosis in the α1^r/r^α2^s/s^ mouse failed to do so in the α1^s/s^α2^s/s^. Taken together, these results are indicative of a link between circulating CTS, Na/K-ATPase α1, ROS, and physiological cardiac hypertrophy in mice under baseline laboratory conditions.

## 1. Introduction

Levels of endogenous cardiac steroids (CTS) vary in response to physiological and pathophysiological stresses such as exercise, pregnancy [1,2,3], hypertension [2,4,5], and heart failure [2,6]. The role of endogenous CTS as hormones with distinct but related modulatory effects on growth as well as cardiovascular equipoise [7,8,9] and dysfunction [5,6,10,11,12] has been increasingly recognized. In the meantime, key issues such as their respective contributions, synthetic pathways, and regulations are areas of very active investigation [9,12,13,14,15,16,17].

CTS effects are mediated through the Na^+^/K^+^-ATPase (NKA), the plasma membrane transporter that utilizes the energy from ATP hydrolysis to catalyze the exchange of intracellular Na^+^ for extracellular K^+^ [18,19]. In the pharmacological-to-toxic range, CTS inhibit the NKA enzyme. The adjustment of Na^+^/Ca^2+^ exchange that ensues ultimately leads to the modulation of cardiac contractility and excitability, which is the molecular basis for the use of the CTS digoxin in the clinical management of heart failure and atrial fibrillation [19,20,21,22].

Understanding the physiological/pathophysiological role of CTS/NKA interaction beyond the effect of administration of exogenous CTS in the therapeutic-to-toxic range has been more challenging. In particular, it has been difficult to establish that systemic concentrations of endogenous CTS reach levels that significantly inhibit NKA [2,23,24,25,26]. Modulation of the more recently discovered, non-canonical NKA receptor signaling function has emerged as a more likely mechanism of action for endogenous CTS [27,28,29]. Indeed, CTS concentrations as low as 100 times below the inhibitory threshold have been shown to induce the assembly of multiple protein complexes into functional microdomains and activate diverse signaling pathways [30]. In the ensuing NKA signaling cascades, activation of Src, 1,4,5-triphosphate receptor (IP3R), EGFR, mitogen-activated protein kinases, mitochondrial reactive oxygen species (ROS), and intracellular Ca^2+^ oscillations has been reported in many cells and organs [9,31,32].

Early experimental evidence supporting structural changes of cardiac cells in response to CTS were reported by Peng et al. and Huang et al. [33,34]. Subsequent studies have exposed the role of ROS in this process [24,31,35]. This line of work, expanded to multiple systems and in vivo models, has led to the current understanding of the Na/K-ATPase/Src/ROS feedforward mechanism and its modulation by CTS [36]. One of the most direct evidence for the importance of this loop in cardiac tissue came from a study by Liu et al., in which blockade of the loop using pNaKtide injection dampened the hypertrophic remodeling of uremic cardiomyopathy in vivo [37]. However, because those studies used pharmacological approaches to inhibit the pathway, non-specific/off-target effects have not been ruled out. To obtain a direct evidence that interaction between endogenous CTS and Na/K-ATPase regulates cardiac ROS and hypertrophic growth, we here compare the cardiac phenotype of mice expressing the rodent wild type Na/K-ATPase α1 with a low affinity for CTS to that of mice genetically engineered to express a Na/K-ATPase α1 with high affinity for CTS. This study, which is the first to examine the CTS-sensitive α1^s/s^α2^s/s^ mouse heart, reveals a mild but significant cardiac phenotypic change consistent with a model whereby increased CTS binding to NKA increases cardiac ROS, favors physiological hypertrophic growth, and reduces angiotensin II-induced fibrosis. RNA sequencing reveals an upregulation of metabolic pathways, especially fatty acid β-oxidation, and a downregulation of pathways related to the extracellular matrix organization in the α1^s/s^α2^s/s^ mouse heart.

## 2. Results

### 2.1. Cardiac Na^+^/K^+^-ATPase in the α1^s/s^α2^s/s^ Mouse

**Figure 1:**
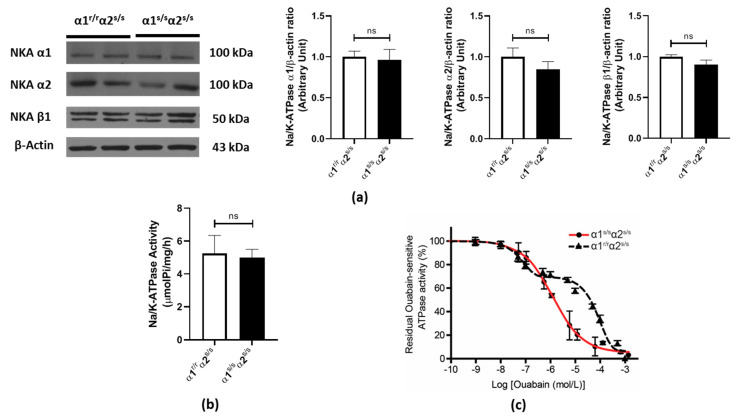
Increased Na/K-ATPase sensitivity to ouabain in α1^s/s^α2^s/s^ hearts: (**a**) Representative Western blots and quantitative analysis for Na/K-ATPase α1, α2, and β1 isoforms in heart homogenates (*n* = 5–6 hearts/genotype). (**b**) Maximal ATPase activity in crude membrane fractions was measured using a colorimetric assay for Pi release (*n* = 6 hearts/genotype). (**c**) Na/K-ATPase dose–response curve to the cardiotonic steroids (CTS) ouabain. Maximal ATPase activity in crude membrane fractions was measured using a colorimetric assay for Pi release in the presence of the ionophore alamethicin and the indicated concentrations of ouabain. The monophasic curve obtained for the α1^s/s^α2^s/s^ is shown in red along with the biphasic curve obtained in the wild-type (α1^r/r^α2^s/s^) mouse heart (*n* = 3 hearts/genotype). ns: non-significant, *p* > 0.05.

To assess whether increasing sensitivity to CTS affected Na/K-ATPase isoform expression and enzymatic activity, we compared heart preparations from α1^s/s^α2^s/s^ mice and α1^r/r^α2^s/s^ littermates. Expression levels of cardiac Na/K-ATPase subunits (α1, α2 and β1) and total enzymatic activity were not altered (Figure 1a,b). Na/K-ATPase in the mouse heart typically exhibits a biphasic dose–response curve to the CTS ouabain, with a low affinity site attributable to α1-containing isoenzymes and a high affinity component attributable to α2-containing isoenzymes. In contrast, dose–response curves from α1^s/s^α2^s/s^ hearts were best fitted assuming one IC_50_, resulting in a monophasic curve and a computed IC_50_ around 10^−6^ M (Figure 1c). This leftward shift in ouabain dose–response is consistent with the expected impact of R111Q and D122N substitutions on NKA α1, which resulted in greater sensitivity and made it indistinguishable from the α2 site in α1^s/s^α2^s/s^ mice.

### 2.2. Cardiac Phenotype of the α1^s/s^α2^s/s^ Mouse

**Figure 2:**
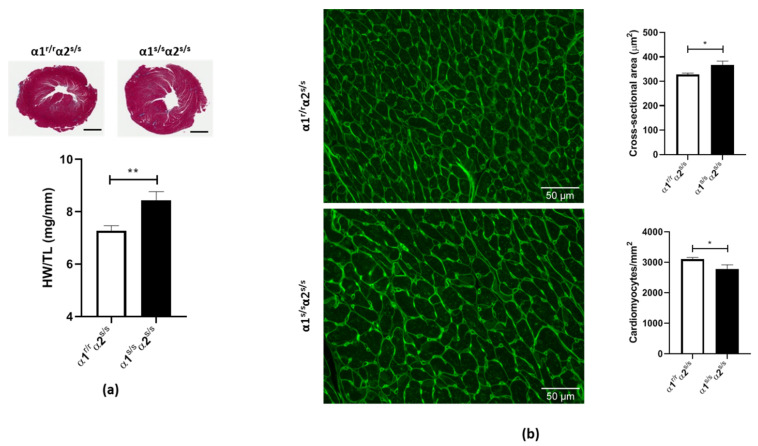
Cardiac hypertrophy in α1^s/s^α2^s/s^ mice: (**a**) Cross-section of whole left ventricles stained with Masson’s trichrome (top); scale bar = 1 mm. Heart weight/tibia length (HW/TL) quantification (bottom) (*n* = 14–16 hearts/genotype). (**b**) Wheat germ agglutinin (WGA) staining of left ventricle cross-sections from α1^r/r^α2^s/s^ (top left) and α1^s/s^α2^s/s^ (bottom left) mice; scale bar = 50 μm. Average cardiomyocyte cross-sectional area (top right) was automatically quantified in over 1000 cells from at least five different random fields (*n* = 4–7 hearts/genotype). Cardiomyocyte density (bottom right) was determined by the number of cardiomyocytes per mm^2^. * *p* < 0.05; ** *p* < 0.01.

A moderate but significant increase in cardiac weight in α1^s/s^α2^s/s^ hearts was observed, as shown in Figure 2a (15% increase in HW/TL ratio, *p* < 0.01). Since increased HW/TL ratios are commonly associated with increased cardiomyocyte size, we next evaluated cardiomyocyte cross-sectional area (CSA) and density in left ventricle histological preparations stained with wheat germ agglutinin (WGA). A significant increase of 15% of the mean cardiomyocyte area was detected in α1^s/s^α2^s/s^ compared to α1^r/r^α2^s/s^ littermates (Figure 2b). Accordingly, a decrease in cardiomyocyte density was observed due to the increased cardiomyocyte size (Figure 2b).

### 2.3. Absence of Adverse Cardiac Remodeling in α1^s/s^α2^s/s^ Mice

**Figure 3:**
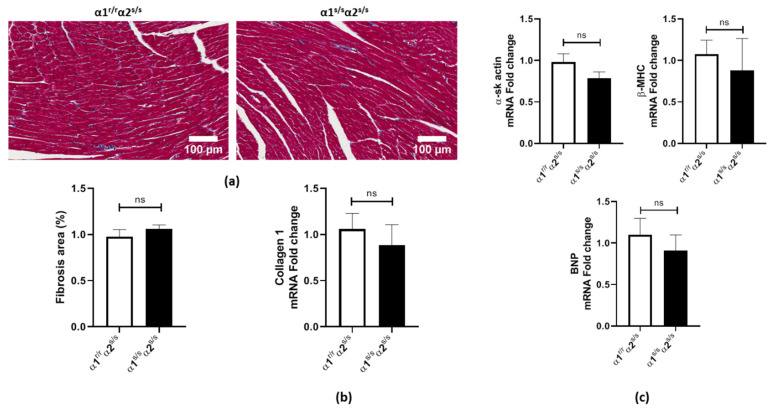
Absence of cardiac fibrosis or change in the fetal gene program in α1^s/s^α2^s/s^ mice: (**a**) Representative histological sections of left ventricles from α1^r/r^α2^s/s^ and α1^s/s^α2^s/s^ (top) mice stained with Masson’s trichrome; scale bar = 200 μm. Fibrosis area quantification (bottom) (*n* = 5 hearts/genotype). (**b**) Collagen-1 mRNA levels in the left ventricle determined by RT-qPCR (*n* = 4–5 hearts/genotype). (**c**) mRNA levels of α-skeletal actin (α-sk actin), β-heavy myosin chain (β-MHC), and brain natriuretic peptide (BNP) in left ventricles determined by RT-qPCR (*n* = 6 hearts/genotype). ns: non-significant, *p* > 0.05.

Physiological stimuli such as exercise result in cardiac hypertrophy, which is characterized by an overall normal cardiac structure and the absence of adverse remodeling, preserved or improved cardiac function, and minimal alteration in cardiac gene expression. In contrast, pathological hypertrophy is associated with adverse remodeling, functional alteration, and/or re-expression of fetal genes. Accordingly, we set out to assess cardiac fibrosis and re-expression of the fetal gene program in α1^s/s^α2^s/s^ mice. No evidence of fibrosis was observed by Masson’s trichrome staining (Figure 3a). Collagen-1 mRNA content was also found to be unchanged compared to the control littermates (Figure 3b). The cardiac fetal gene program, a key feature of pathological cardiac hypertrophy, was not changed in α1^s/s^α2^s/s^ compared to α1^r/r^α2^s/s^ littermates (Figure 3c).

Furthermore, we analyzed the systolic function of α1^s/s^α2^s/s^ mice by echocardiography. We observed a significant increase in the left ventricle mass and left ventricle anterior wall thickness, at systole, in agreement with the histological characterization (Table 1). Despite those changes, no major changes in systolic cardiac function were observed. Given the absence of fibrosis, fetal gene program re-expression, and functional abnormalities, it was concluded that increased α1 affinity for endogenous CTS had favored physiological cardiac hypertrophy in this gain-of-function mouse model.

### 2.4. RNA-Seq Analysis

**Figure 4:**
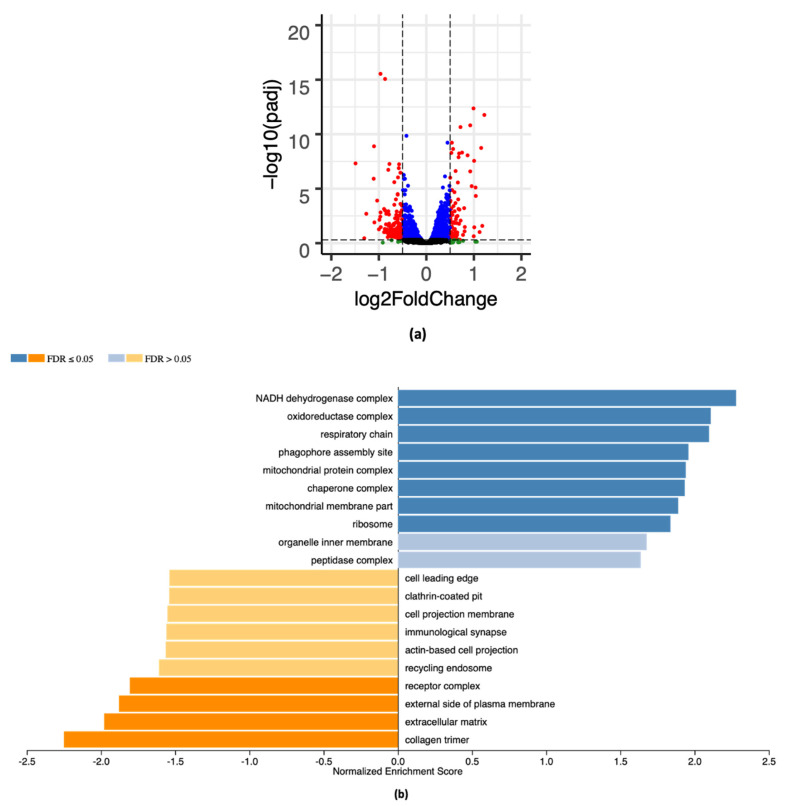

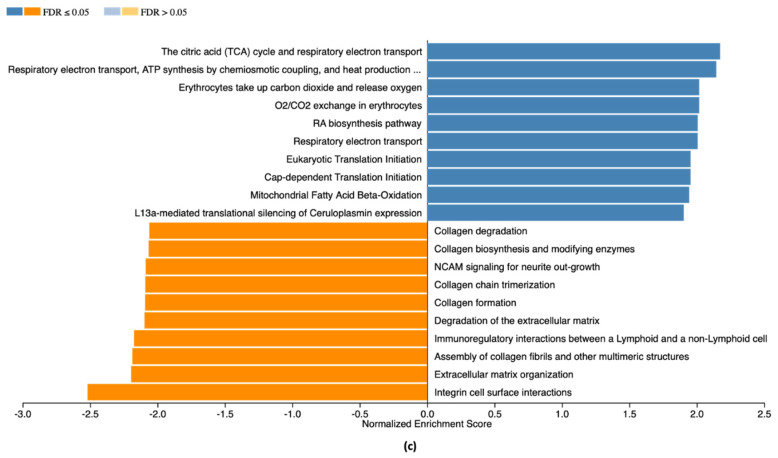
RNA-seq analysis comparing the transcriptome of α1^r/r^α2^s/s^ and α1^s/s^α2^s/s^ hearts: (**a**) Volcano plot of gene expression in α1^r/r^α2^s/s^ and α1^s/s^α2^s/s^ hearts plotting -log10 of adjusted *p*-value on y-axis and log2 fold change on x-axis. Red dots are downregulated (left) and upregulated (right) genes with a log2 fold change greater than 0.5. Blue dots represent those genes with a log2 fold change less than 0.5. Green and black dots represent genes with an adjusted *p*-value greater than 0.05. (**b**) The enriched pathways in the category of Cellular Component of the Gene Ontology database. (**c**) Pathway enrichment using the Reactome database. Pathway enrichment analysis were performed with Gene Set Enrichment Analysis (GSEA) as described in the Materials and Methods section. Blue bars represent upregulated pathways and orange bars represent downregulated pathways.

RNA-seq analysis was carried out in whole hearts from 3-month-old α1^s/s^α2^s/s^ male mice and age-matched α1^r/r^α2^s/s^ (3 mice/genotype). A volcano plot is shown in Figure 4a with the distribution of genes that were up- (right) or downregulated (left). A total of 388 genes were found to be differentially expressed, 152 being downregulated and 235 upregulated. A Gene Set Enrichment Analysis (GSEA) was performed to assess the pathways that were up- and downregulated in α1^s/s^α2^s/s^ hearts compared to α1^r/r^α2^s/s^ hearts. Using the Cellular Component in the Gene Ontology database (Figure 4b), we observed downregulated pathways, such as the extracellular matrix and collagen trimer. Moreover, upregulated pathways, such as the NADH dehydrogenase complex, respiratory chain, and mitochondrial protein complex, suggested an increase in energy demand that is commonly observed with cardiac hypertrophy [38,39,40,41,42]. Using the Reactome database (Figure 4c), a similar trend was noted for downregulated pathways involving the extracellular matrix, and upregulated pathways related to energy production, such as citric acid cycle, respiratory electron transport, and mitochondrial fatty acid β-oxidation.

### 2.5. Evidence of Activation of the Na/K-ATPase Oxidant Amplification Loop in α1^s/s^α2^s/s^ Mice

**Figure 5:**
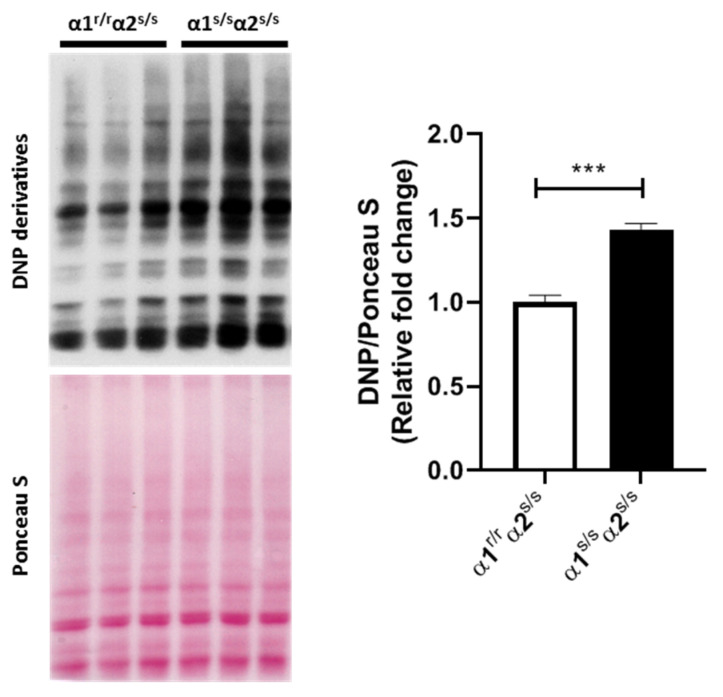
Increased protein carbonylation in α1^s/s^α2^s/s^ hearts: Representative Western blot analysis of protein carbonylation and Ponceau S staining as a loading control in left ventricle homogenates and associated quantitative data (*n* = 7–8 hearts/group). *** *p* < 0.001; DNP: 2,4-dinitrophenyl.

The increased energetic metabolism suggested by RNA-seq analysis is consistent with hypertrophic growth, but also increased the levels of reactive oxygen species (ROS) [43,44,45]. Consistently, increased sensitivity to CTS would be expected to stimulate the NKA α1 oxidant amplification loop [36,46], which prompted us to explore redox signaling in α1^s/s^α2^s/s^ mouse hearts. Indeed, multiple lines of evidence suggest that Na/K-ATPase and ROS induce cardiac hypertrophic growth through close and reciprocal regulatory mechanisms in cultured cardiac myocytes [31,35]. To this end, carbonylated protein content was measured as one of the earliest and more stable indicators of ROS generation. Consistent with an activation of ROS signaling, a 50% increase in protein carbonylation was observed in α1^s/s^α2^s/s^ mice hearts compared to their α1^r/r^α2^s/s^ littermates (Figure 5).

### 2.6. Angiotensin-II Does Not Induce Cardiac Fibrosis in α1^s/s^α2^s/s^ Mice

**Figure 6:**
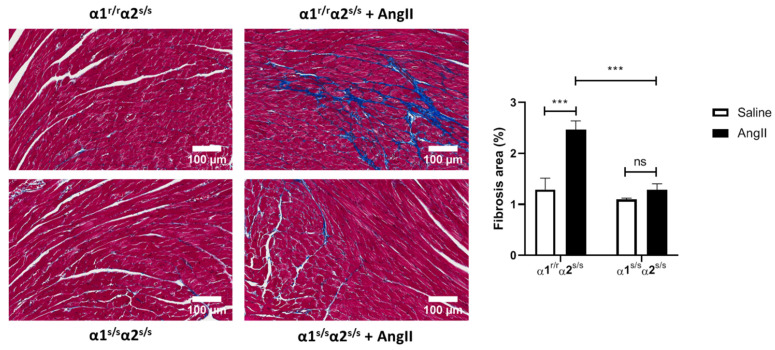
Angiotensin-II does not induce cardiac fibrosis in α1^s/s^α2^s/s^ mice: The left panels show representative histological sections stained with Masson’s trichrome, and the right panels show the quantitative data of fibrosis analyzed using ImageJ (*n* = 4–8 hearts/group). Scale Bar = 100 µm. *** *p* < 0.001; ns: non-significant, *p* > 0.05.

Angiotensin-II (AngII) infusion is a well-known trigger of cardiac fibrosis [47,48]. Since our RNA-seq analysis showed downregulation of pathways involved in extracellular matrix organization, we treated α1^s/s^α2^s/s^ mice and age-matched α1^r/r^α2^s/s^ for two weeks as described in the Materials and Methods section and evaluated cardiac fibrosis. As observed in Figure 6, AngII induced cardiac fibrosis in control α1^r/r^α2^s/s^ mice but failed to do so in α1^s/s^α2^s/s^ mice.

## 3. Discussion

In the 20 years that have elapsed since the first report of ouabain-induced hypertrophic growth in cultured cardiac myocytes, a lot has been learned about the underlying signaling mechanism involved. In the present study, we used the α1^s/s^α2^s/s^ mouse model for the first time to understand the physiological/pathophysiological role of the endogenous CTS/NKA interaction on cardiac structure and function. We observed that a hundred-to-thousand-fold increase in NKA α1 affinity for CTS produces a 15% increase in cardiac mass (Figure 2) in α1^s/s^α2^s/s^ male mice aged 3–6 months with no apparent change in cardiac function (Table 1). This increase in cardiac mass was not a consequence of changes in NKA isoform expression or altered Na^+^/K^+^-ATPase activity in the cardiac muscle (Figure 1), which is consistent with previous studies showing that mutations of amino acids arginine 111 and aspartate 122 of the NKA α-polypeptide do not alter enzyme function or expression in multiple tissues [49,50,51,52,53]. In the “SWAP” model (α1^s/s^α2^r/r^), mice did not have altered cardiac NKA either, but in that model the hundred-to-thousand-fold increase in NKA α1 affinity for CTS did not result in a significant change of cardiac mass when animals were maintained in normal laboratory conditions [50,51]. Certainly, differences in background (mixed 129SvJ /Black Swiss vs. C57Bl6) could contribute to the difference between the cardiac phenotypes of α1^s/s^α2^r/r^ vs. α1^s/s^α2^s/s^ at baseline. Alternatively, the increase in cardiac size observed in the α1^s/s^α2^s/s^ and not in α1^s/s^α2^r/r^ could indicate that CTS binding to both α1 and α2 isoforms contributes to the hypertrophic response.

Our RNA sequencing data (Figure 4) indicated a significant downregulation of pathways related to extracellular matrix organization (e.g., extracellular matrix and collagen trimer in Figure 4b, collagen formation and extracellular matrix organization in Figure 4c). This is in agreement with the absence of fibrosis observed at baseline (Figure 3) and was corroborated functionally by the observation that angiotensin-II challenge does not induce cardiac fibrosis in α1^s/s^α2^s/s^ mice (Figure 6). Furthermore, upregulation of pathways related to metabolism and energy production (e.g., NADH dehydrogenase complex and respiratory chain in Figure 4b; tricarboxylic acid (TCA cycle), respiratory electron transport, ATP synthesis, and mitochondrial fatty acid β-oxidation in Figure 4c) is consistent with increased energy demand in cardiac hypertrophy [38,40,42]. In fact, studies have demonstrated that mice with cardiac-restricted constitutive activation of PI3K exhibit physiological cardiac hypertrophy and increased cardiac fatty acid oxidative capacity [54,55,56].

The results in Figure 5 show a clear increase in cardiac protein carbonylation in the α1^s/s^α2^s/s^ mouse. This suggests that a ROS-related mechanism is involved, which is consistent with the well-established role of ROS in hypertrophic growth of the cardiac myocyte [57,58,59]. Based on multiple evidence of CTS-induced ROS production and subsequent impact on cardiomyocyte growth reported in vitro and in vivo [35,60,61,62], a direct effect of CTS on NKA α1 with increased affinity in the cardiac myocyte most likely played a role in the observed hypertrophy. Although beyond the scope of the present study, future studies may clarify the role of Src-mediated ROS amplification. Moreover, possible roles of NKA α1 related to intracellular ion homeostasis in cardiac myocytes, in other cardiac cell types and/or extra cardiac tissues cannot be excluded, and may have ultimately contributed to the cardiac phenotype of this global α1^s/s^α2^s/s^ mouse model. Indeed, CTS/NKA interactions occur on a systemic level, with multiple opportunities for cross-talks and feedback mechanisms with neurohumoral regulators of cardiovascular function in health and disease [63,64,65]. For example, there is evidence that CTS secretion by adrenocortical cells is stimulated by angiotensin II and the adrenocorticotropic hormone (ACTH) [53,66].

Correlative as well as direct evidence for CTS-induced cardiac remodeling has been reported in both physiological and pathological hypertrophy. Examples of the former include normal postnatal growth [67,68], pregnancy-induced growth [69], and exercise-induced cardiac hypertrophy [1,70]. Evidence for the latter has been shown in chronic pressure or volume overload such as hypertensive disease [14,71,72], post-MI remodeling, and heart failure [11,73,74], as well as in major forms of cardiomyopathies [9,12,75]. Several lines of evidence suggest that the cardiac hypertrophic growth observed in the α1^s/s^α2^s/s^ mouse is physiological rather than pathological in nature. First, the relatively mild increase in cardiac mass observed in the α1^s/s^α2^s/s^ mouse (10%) is in the range typically observed for physiological hypertrophy, rather than the more pronounced effect of pathological hypertrophy induced by pressure overload with transverse aortic constriction (TAC) [76,77,78]. This observed increase in heart size resulted from an increase in myocyte size and occurred without a detectable change in fibrosis. Finally, echocardiographic analysis did not reveal any cardiac dysfunction and RT-qPCR analysis showed that there was no re-expression of the cardiac fetal gene program characteristic of pathological hypertrophy. Therefore, although increases in circulating amounts of CTS have been detected in both types of hypertrophy, the present study suggests that their direct impact in normal laboratory conditions favors the development of the physiological type. In all likelihood, the underlying mechanism involves the PI3K-IA pathway, which is typically associated with physiological cardiac hypertrophy, and is initiated upon exposure of cardiac myocytes to the CTS ouabain in vitro and in vivo. On the other hand, the cardiac phenotypic changes of the α1^s/s^α2^s/s^ mouse do not reflect the well-known impact of the CTS/NKA α1 pathway involved in the development of fibrosis observed in vitro and in vivo [12,16,37,79,80,81] and in adverse remodeling associated with pathological hypertrophy [12,63,64,79,81,82,83]. This may be an additional indication that various endogenous CTS exert complementary and antagonistic effects, possibly through biased signaling [84,85,86] on cardiac structure. Such biased molecular mechanisms have been proposed in the regulation of blood pressure by CTS [87,88,89]. Therefore, the nature and amount of circulating CTS in health and diseases may ultimately dictate the type of hypertrophic response. In addition, crosstalk with various pathways that affect the NKA receptor itself, such as the deleterious ROS amplification loop encountered in uremic cardiomyopathy, is likely to alter the effects of CTS. Specifically, the stability of the α1 isoform expression combined with low basal levels of endogenous CTS in the α1^s/s^α2^s/s^ mice, in contrast to decreased NKA α1 expression levels and elevated CTS in disease models such as cardiac hypertrophy, heart failure, and cardiomyopathy [90,91,92,93,94,95] may explain the occurrence of physiological vs. pathological hypertrophy through the CTS/NKA receptor.

## 4. Materials and Methods

### 4.1. Materials

Anti Na/K-ATPase α1 (NASE) primary-antibody was a gift from Drs. T. A. Pressley and P. Artigas, Texas Tech University HSC, Lubbock, TX, USA. Anti Na/K-ATPase α2 (AB-9094-I), and anti Na/K-ATPase β1 (05-382) were from Millipore (Billerica, MA, USA). β-actin (sc-7210), rabbit, and mouse secondary antibodies (sc-2004 and sc-2005, respectively) were purchased from Santa Cruz Biotechnology (Dallas, TX, USA). The 2,4-Dinitrophenylhydrazine (DNPH, D199303) and antibody against 2,4-dinitrophenyl (DNP, D9656) hydrazone derivatives were from Sigma-Aldrich (St. Louis, MO, USA).

### 4.2. Generation of the NKA α1 Sensitive Mouse Model

Mice expressing Na/K-ATPase (NKA) α1 isoform with high-affinity for CTS were created by introducing R111Q and D122N substitutions in the mouse α1 isoform of Na/K-ATPase [49]. Mice obtained from established colonies at the University of Cincinnati were backcrossed to a C57Bl6 background. Males NKA α1 sensitive (α1^s/s^α2^s/s^) and their control littermates (α1^r/r^α2^s/s^) aged 3 to 6 months were used in this study. Genotyping was performed using the following primers: A1Bgl-30 forward (5′-GAC ATG CAA AAC CGA ACC AG-3′) and A1bgl+164 reverse (5′-GGA GAT GAC AAG GTC CAG GG-3′).

All animals were kept in a 12-h dark/light cycle and fed standard chow *ad libitum*. All animal care and experiments were approved by the Marshall University Institutional Animal Care and Use Committee (IACUC) in accordance with the National Institutes of Health (NIH) Guide for the Care and Use of Laboratory Animals (NIH Publication, 8th Edition, 2011).

### 4.3. Western Blot Analysis

Whole heart lysates were prepared by homogenization in RIPA buffer as described [96]. Equal amounts of proteins were loaded and separated by 10% SDS-PAGE, transferred onto nitrocellulose membrane (GE Healthcare Life Sciences, Pittsburgh, PA, USA), and probed with the primary antibody (anti-NKA α1 1:1000; anti-NKA α2 1:1000; anti-NKA β1 1:1000; anti-β-actin 1:1000) followed by incubation with the HRP-conjugated secondary antibody. The signal was detected using chemiluminescence, and quantified using Image J software (National Institutes of Health (NIH), Bethesda, MD, USA). Cardiac protein carbonylation was assessed as previously described [97] using an anti-DNP antibody (dilution 1:20,000).

### 4.4. Na/K-ATPase Activity and Ouabain Dose-Response in Heart Lysates

Hearts were snap frozen in liquid nitrogen immediately after harvesting. Powdered heart tissue was placed into 10 mL ice-cold 1M KCl and homogenized in a 30 mL homogenizer by 15 times up-and-down strokes and followed by homogenization with a tissue mixer homogenizer at 30,000 rpm for 60 s (twice, 30 s each). The homogenates were centrifuged at 100 g for 10 min to remove cellular debris and unbroken cells. The supernatants were centrifuged at 1000 g for 10 min. The sediment was washed once with a solution containing 50 mM KCl and 50 mM Tris-HCl (pH 7.4), and twice with 50 mM Tris-HCl (pH 7.4). The final pellet was resuspended in 1mM Tris-EDTA (pH 7.4). The resulting crude homogenates were incubated with the ionophore alamethicin (0.1 mg/mg protein) 10 min at 37 °C prior to ouabain sensitive ATPase activity measurement [98]. This alamethicin treatment is necessary to ensure the access of substrates and inhibitors to both the ATP- and ouabain-binding sites of the enzyme in closed membrane vesicles that may form in crude homogenates. Na/K-ATPase activity was measured in alamethicin-pretreated samples (50 µg protein/sample) by colorimetric determination of inorganic phosphate released after incubation of 10 min at 37 °C in a reaction buffer containing (in mmol/L) Tris-HCl (20), MgCl_2_ (1), NaCl (100), KCl (20), EGTA-Tris (1), and NaN_3_ (5). After addition of 2 mmol/L Mg^2+^/ATP, the enzymatic reaction was allowed to run for 10 min before the addition of 1 mL ice-cold 8% trichloroacetic acid to terminate the reaction. The amount of phosphate released was determined using an inorganic phosphate detection kit (AK-111, Biomol Research Laboratories, Inc., Plymouth Meeting, PA, USA), according to the manufacturers’ recommendation. Ouabain insensitive activity was measured in a separate reaction in the presence of 2 mmol/L ouabain in the same buffer. Ouabain sensitive Na/K-ATPase activity was then determined by subtracting ouabain insensitive from total ATPase activity. To establish the ouabain dose–response curve, control hearts tissue was prepared as described above, and indicated concentrations of ouabain were added in the reaction buffer. The resulting data were analyzed by nonlinear regression and best fitted with biphasic curve or monophasic curve using GraphPad Prism 5.0 (GraphPad Software, Inc, La Jolla, CA, USA).

### 4.5. Echocardiography

Echocardiography was carried out under light anesthesia (1–2% isoflurane in oxygen) placed on a heating pad using a MS400: 18–38 MHz operating frequency MicroScan transducer and the Vevo 1100 Imaging System. Left ventricle dimensions were obtained during TM mode acquisition from the parasternal short axis view at the level of the papillary muscles. Images were analyzed with the Vevo 1100 Imaging System software (FUJFILM VisualSonics Inc., Tokyo, Japan), by an examiner blinded to the genotype of the animals as described [37].

### 4.6. Histology

Hearts were fixed in 10% buffered formalin solution and stored in 70% EtOH at 4 °C until embedding. Paraffin embedding, transverse sectioning, Wheat Germ Agglutinin (WGA) immunostaining, Masson’s trichrome staining, and imaging were performed by Wax-it Histology Services Inc. (Vancouver, BC, Canada). Dehydration was performed with ethanol 70%, 95% (2×) and 100% (3×) for 2 h, each followed by xylene (3×) for 1 h each. The specimens were then impregnated with paraffin at 60 °C (3 successive changes after 45 min, 1 h, 2 h under vacuum) and then embedded into wax blocks.

Masson’s trichrome staining was performed to assess cardiac fibrosis. Sections were rehydrated using xylene, decreasing % of ethanol, and water. Sections were treated with saturated picric acid at 60 °C for 1.5 h, rinsed, and stained in Weigart’s hematoxylin solution for 30 min. After rinsing, they were treated with acetic acid for 30 s, rinsed, stained in acid fuchsin–ponceau xylidine for 2 min, and rinsed. They were then treated in 1% phosphomolybdic acid for 5 min and stained with 1% aniline blue for 15 min. prior to rinsing with successive changes of 1% acetic acid and water. Total (i.e., interstitial plus perivascular) fibrosis was determined in each sample using 10 randomly selected fields from a 20× magnification whole heart scan and analyzed using ImageJ software (RSB NIH), which allows quantification of the positive blue stained area (fibrosis) and total area [99]. Fibrosis was expressed as percent of total area.

WGA immunostaining was used to examine cardiomyocyte cross-sectional area and density. Immunostaining was performed following rehydratation of sections in Tris buffered saline pH 7.4, followed by antigen retrieval (citrate buffer in steamer for 25 min) and permeabilized with TBS-T (0.5%) for 20 min at room temperature. Protein blocking was performed for 30 min at room temperature. WGA at a 1:200 dilution was incubated overnight at 4 °C, followed by secondary antibody tagged with Alexa 568 at a 1/500 dilution (30 min at room temperature). Sections were mounted with DAPI-Prolong gold (Invitrogen). To assess cardiomyocyte cross-sectional area and density, 10 fields per left ventricle stained by WGA were selected using a 40× magnification. The number and area of cardiomyocytes were determined using an ImageJ macro developed by Dr. Kees Straatman at the University of Leicester for automated batch processing of the images. The mean cardiomyocyte area and density was measured in over 1000 cells from at least 4 hearts per genotype.

### 4.7. Determination of mRNA Levels

Total RNA was extracted from cardiac tissue using Trizol^®^ reagent according to the manufacturer’s instructions (Life Technologies, Carlsbad, CA, USA). The amount and quality of extracted RNA were assessed using the Nanodrop 2000 (Thermo Scientific, Waltham, MA, USA). First-strand cDNA was synthesized from mRNA using Superscript II First-Strand system (Invitrogen, Carlsbad, CA, USA). Gene expression of collagen 1, brain natriuretic peptide (BNP), alpha skeletal actin (α-sk actin), and beta myosin heavy chain (β-MHC) was analyzed by real-time quantitative PCR using PowerSYBR green (Life Technologies, Carlsbad, CA, USA). Real-time quantitative PCR was performed using a LightCycler^®^ 480 Instrument II (Roche, Indianapolis, IN, USA) in a 384-well plate. Melting curve analysis was performed to ensure purity of the PCR products, and relative quantification was determined using the comparative CT method with data normalized to GAPDH and calibrated to the average of control group, as previously described [100]. Primer sequences are listed in Supplemental Table S1.

### 4.8. Angiotensin-II Treatment

Three-month-old male mice were treated with a pressor dose of Angiotensin-II (AngII) of 1.5 mg.kg^−1^.d^−1^ or saline for 14 days via osmotic minipumps (model 2002, AlzaCorp, Palo Alto, CA, USA), implanted subcutaneously at the dorsum of the neck as previously described [101].

### 4.9. RNA Sequencing (RNA-Seq) Analysis

Total RNA was extracted from cardiac tissue using Trizol^®^ reagent (Life Technologies, Carlsbad, CA, USA) and the RNeasy Midi Kit (Qiagen, Hilden, Germany) according to the manufacturers’ instructions. RNA-seq was performed by Novogene (Sacramento, CA, USA) using the Illumina NovaSeq 6000 system. Quality control, mapping to reference genome, and quantification were performed by the company according to their standard protocol. Using the raw count data provided, we performed a differential gene expression (DEG) analysis using the DESeq2 R-package [102]. A Volcano Plot was constructed based on the DEG data using the EnhancedVolcano R-package. The gene list and associated log2 fold change obtained from the DESeq2 analysis were used as the input for Gene Set Enrichment Analysis (GSEA) using the WebGestalt toolkit (www.webgestalt.org. Accessed 17 February 2021.) [103]. The Cellular Component category of Gene Ontology and the Reactome database were selected for pathway enrichment analysis.

### 4.10. Statistical Analysis

The data are presented as means ± SEM. The data were analyzed by Student’s t-test for comparison between two independent groups and by two-way ANOVA followed by Tukey’s multiple comparisons test for grouped analysis. The data were stored and analyzed using GraphPad Prism software (La Jolla, CA, USA). A probability value of *p* < 0.05 was considered statistically significant.

## Figures and Tables

**Table 1:** Echocardiographic parameters in α1^r/r^α2^s/s^ and α1^s/s^α2^s/s^ mice. * *p* < 0.05.

*Parameter*	*α1^r/r^α2^s/s^ (n = 9)*	*α1^s/s^α2^s/s^ (n = 6)*
HR (bpm)	457 ± 23	475 ± 31
LVID; s (mm)	2.50 ± 0.52	2.60 ± 0.42
LVID; d (mm)	3.61 ± 0.54	3.86 ± 0.28
LV Volume; s (µL)	23.7 ± 10.6	25.5 ± 9.2
LV Volume; d (µL)	56.6 ± 18.7	64.9 ± 11.0
SV (µL)	32.9 ± 11.2	39.4 ± 3.8
EF (%)	59 ± 11	62 ± 9
FS (%)	31 ± 7	33 ± 7
CO (mL/min)	14.9 ± 4.7	18.7 ± 2.1
LVAW; s (mm)	1.34 ± 0.16	1.53 ± 0.12 *
LVAW; d (mm)	0.95 ± 0.12	1.06 ± 0.11
LVPW; s (mm)	1.19 ± 0.17	1.24 ± 0.24
LVPW; d (mm)	0.80 ± 0.10	0.84 ± 0.18
LV Mass (mg)	87.1 ± 12.4	110.7 ± 19.9 *

## Data Availability

The data presented in this study have been deposited at the Gene Expression Omnibus at the National Center for Biotechnology Information and may be accessed using the accession number GSE169734.

## Supplementary Materials

The following are available online at https://www.mdpi.com/article/10.3390/ijms22073462/s1, Table S1: Primers used for RT-qPCR.
