# Supplementary material for: Cardiac Oxidative Signaling and Physiological Hypertrophy in the Na/K-ATPase α1s/sα2s/s Mouse Model of High Affinity for Cardiotonic Steroids
Source: Int J Mol Sci. 2021 Mar 27;22(7):3462. doi: 10.3390/ijms22073462 (PMC8036649; doi:10.3390/ijms22073462)
Supplement: Supplementary file 1 [file ijms-22-03462-s001.pdf]

Supplemental Table S1: Primers used for RT-qPCR

Name	Sequence	Gene
**BNP**	**F:** GAG TCC TTC GGT CTC AAG GC<br>**R:** CAA CTT CAG TGC GTT ACA GCC	NM_008726.5
**β-MHC**	**F:** TCC TGC TGT TTC CTT ACT TGC T<br>**R:** GGC TGA GCC TTG GAT TCT CAA A	NM_001164171.1
**α-sk actin**	**F:** ACA CGC CAG CCT CTG AAA CT<br>**R:** CCG TTG TCA CAC ACA AGA GC	NM_009606.3
**Collagen 1**	**F:** CGA TGG ATT CCC GTT CGA GT<br>**R:** CGA TCT CGT TGG ATC CCT GG	NM_007742.4
**GAPDH**	**F:** CTT TGT CAA GCT CAT TTC CTG G<br>**R:** TCT TGC TCA GTG TCC TTG C	NM_008084
